# Supplementary material for: DMRforPairs: identifying Differentially Methylated Regions between unique samples using array based methylation profiles
Source: BMC Bioinformatics. 2014 May 15;15:141. doi: 10.1186/1471-2105-15-141 (PMC4046028; doi:10.1186/1471-2105-15-141)
Supplement: Additional file 2 — DMRforPairs output for the comparison of A431-MCF7 and NA17018-NA17105. Please start from the HTML files in each folder. Available via the BMC Bioinformatics website. [file 1471-2105-15-141-S2.zip › 1394847754114233_MOESM2_ESM/A431_MCF7/figures/20654.pdf]

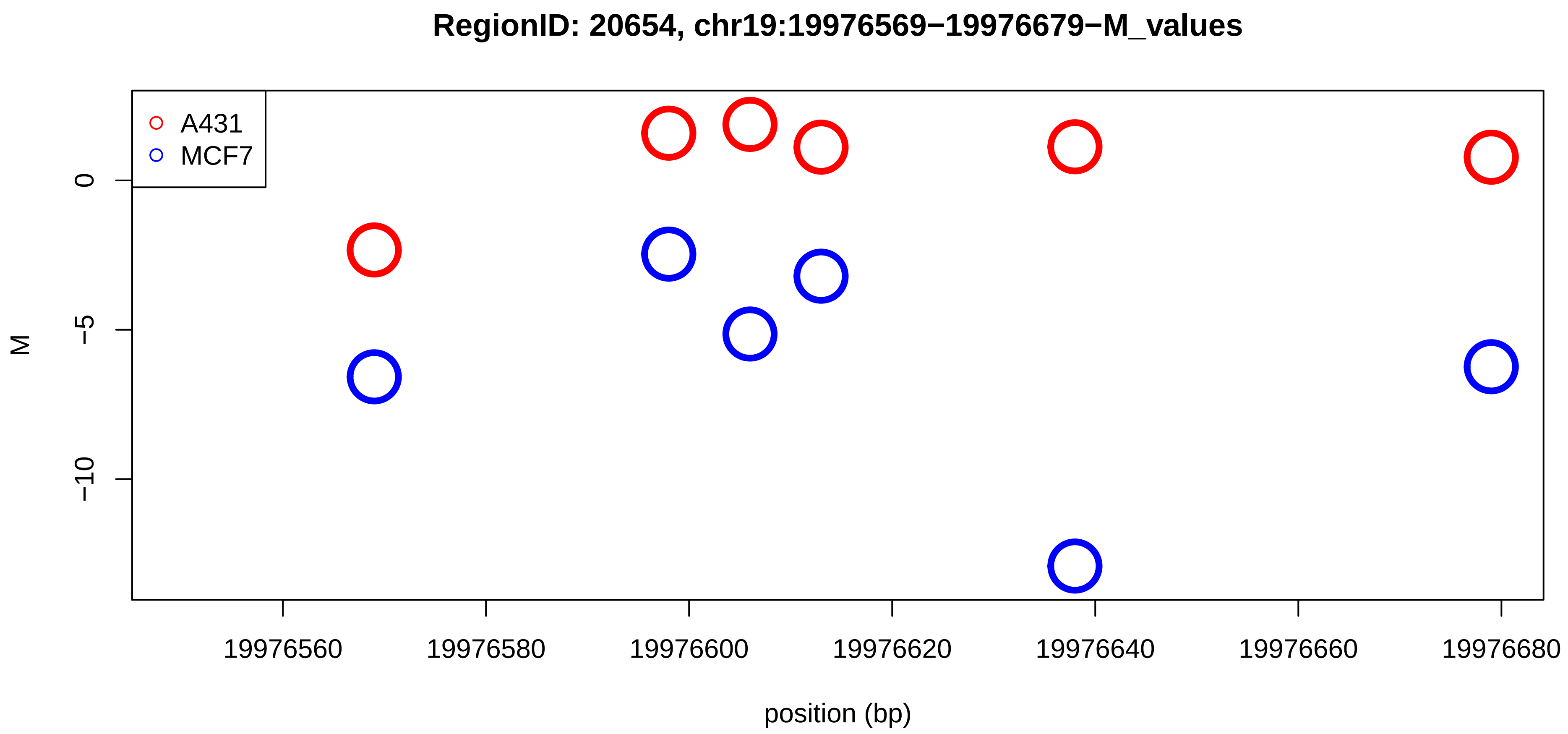
RegionID: 20654, chr19:19976569−19976679−M_values
A431
MCF7
M
0
−5
−10
19976560
19976580
19976600
19976620
19976640
19976660
19976680
position (bp)

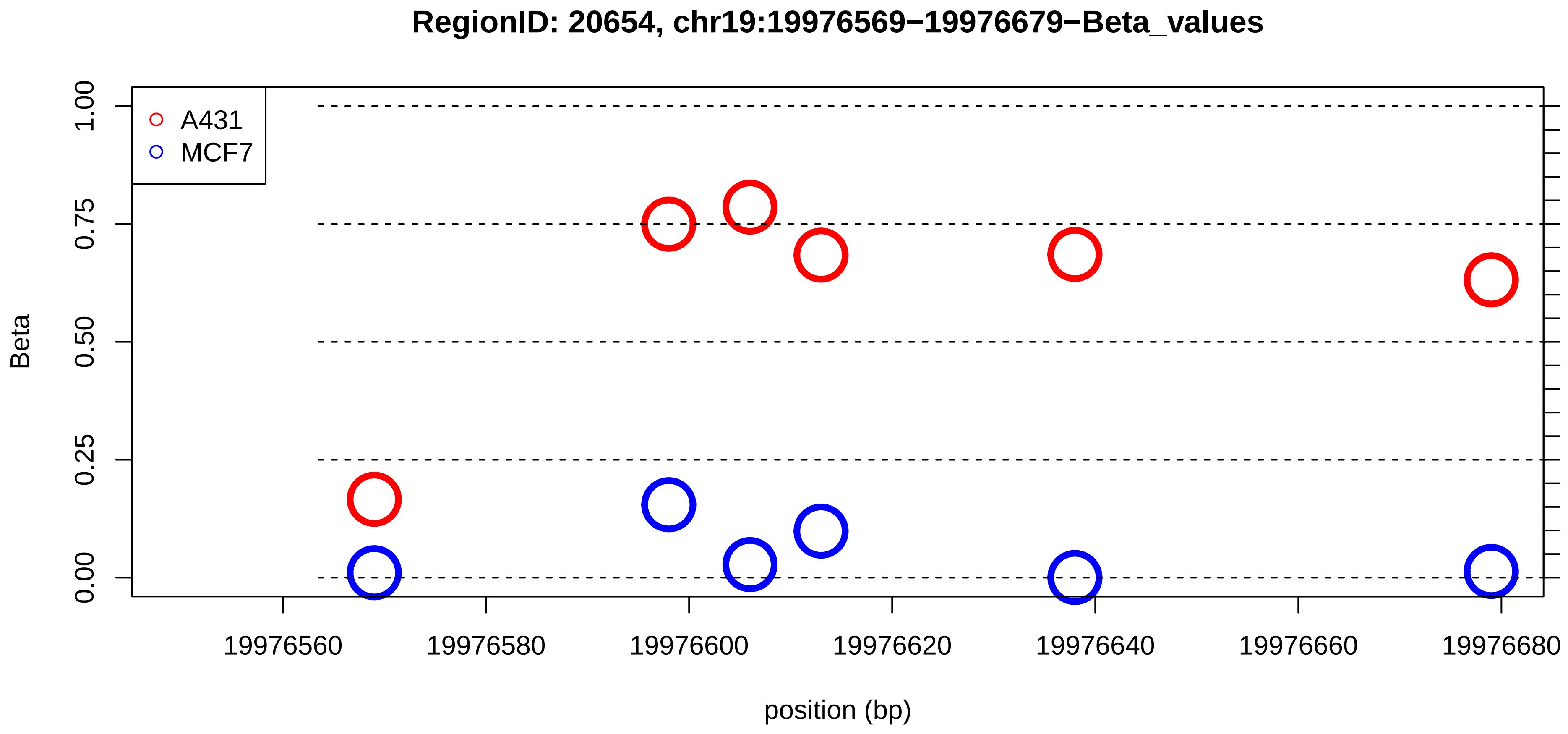
RegionID: 20654, chr19:19976569−19976679−Beta_values
A431
MCF7
Beta
1.00
0.75
0.50
0.25
0.00
19976560
19976580
19976600
19976620
19976640
19976660
19976680
position (bp)

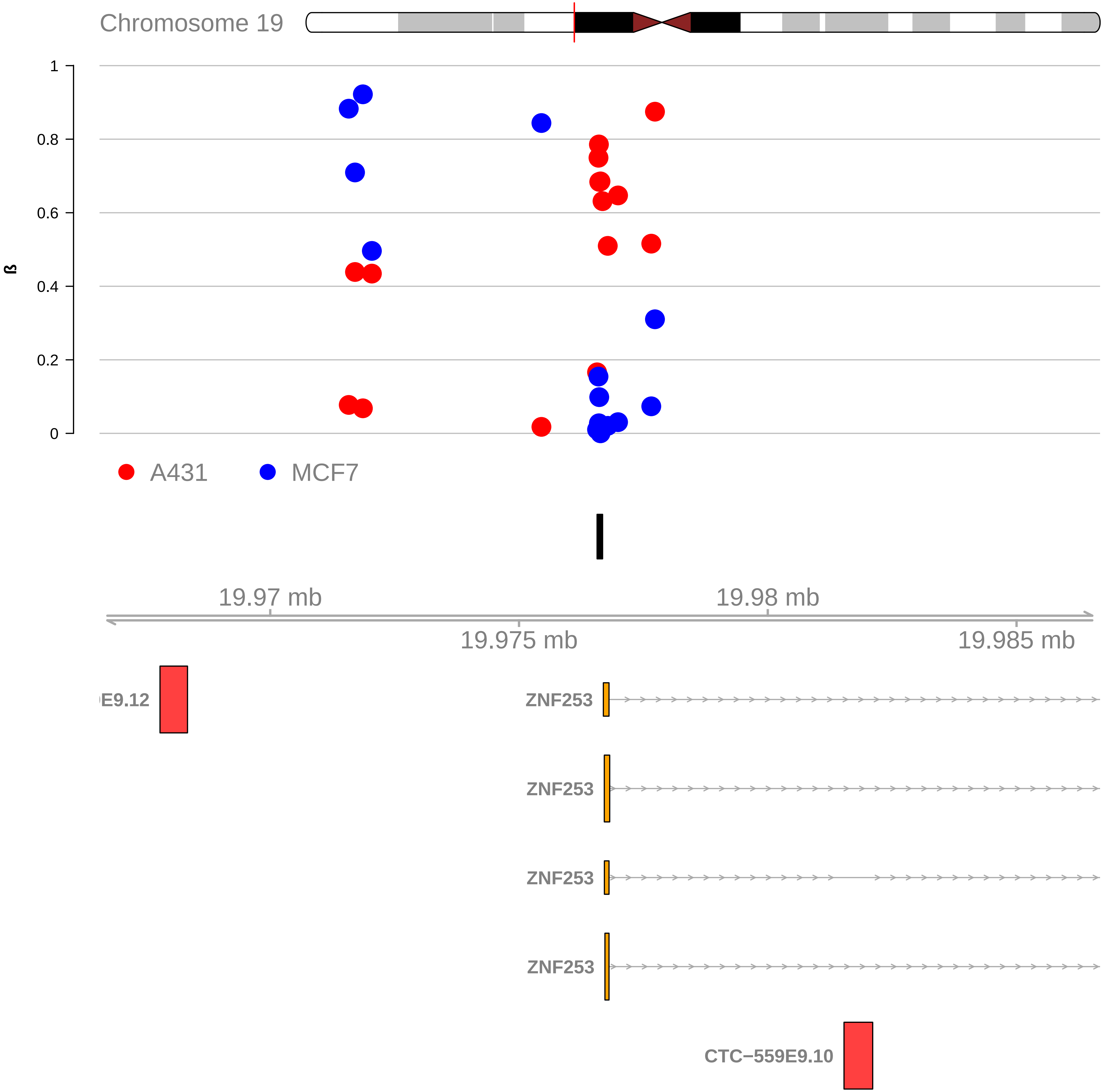

Chromosome 19
1
0.8
0.6
0.4
0.2
0
ß
A431
MCF7
19.97 mb
19.98 mb
19.975 mb
19.985 mb
E9.12
ZNF253
ZNF253
ZNF253
ZNF253
CTC−559E9.10
